# Supplementary material for: Content-rich biological network constructed by mining PubMed abstracts
Source: BMC Bioinformatics. 2004 Oct 8;5:147. doi: 10.1186/1471-2105-5-147 (PMC528731; doi:10.1186/1471-2105-5-147)
Supplement: Additional File 2 — The original results of the above study (non-essential files are deleted to keep the file size under the limit set by BMC bioinformatics). [file 1471-2105-5-147-S2.bz2 › chilibotAdditionalFile2/dip20/217ID7744001E725/html/IL6ST_IL6.html]

 


 **IL6ST** and **IL6** 
  
Found 848 abstracts in PubMed, retrieved 20.  
 

 What does Google say? 
 PDF only 
| .edu only 

---

**Interactive relationship** (e.g. stimulation, inhibition, etc)

**Neutral relationship**- AIMS Interleukin 6  [ **IL6** ]  IL 6  [ **IL6** ]  contributes via its signal transducer gp130  [ **IL6ST** ]  to the acute phase response APR in hepatocytes.  Ref: 12891556 Gastroenterology, 2003
- Ciliary neurotrophic factor CNTF is a gp130  [ **IL6ST** ]  neuroregulatory cytokine belonging to the interleukin 6  [ **IL6** ]  type cytokine superfamily.  Ref: 12890930 Gynecol Obstet InvestGynecol Obstet Invest, 2003
- Thus, constitutive activation of STAT3 in HNSCC may use an autocrine paracrine activating loop mediated by  **IL6**  and other cytokines acting through the gp130  [ **IL6ST** ]  receptor family, which may confer both proliferative and survival potential in this malignancy.  Ref: 12782602 Cancer Res, 2003
- In conclusion, our experiments indicate an involvement of IL 6  [ **IL6** ]  in the pathogenesis of liver diseases and suggest a protective role of IL 6  [ **IL6** ]  gp130  [ **IL6ST** ]  dependent pathways in nonparenchymal liver cells during fibrosis progression in chronic liver diseases.  Ref: 12830005 Hepatology< MedlineTA>Hepatology, 2003
- Interleukin 6  [ **IL6** ]  IL 6  [ **IL6** ]  is an immunoregulatory cytokine that activates a cell surface signaling assembly composed of IL 6  [ **IL6** ] , the IL 6  [ **IL6** ]  alpha receptor IL 6Ralpha, and the shared signaling receptor gp130  [ **IL6ST** ] .  Ref: 12829785 Science, 2003
- The subsequent recruitment of the common signal transducer gp130  [ **IL6ST** ]  and activation of the JAK STAT and SHP 2 Ras mitogen activated protein kinase MAPK pathways are responsible for the majority of cellular responses elicited by IL 6  [ **IL6** ]  cytokines.  Ref: 12764151 J Biol Chem< MedlineTA>J Biol Chem, 2003
- IL 6  [ **IL6** ]  type cytokines exert their action via the signal transducers gp130  [ **IL6ST** ] , LIF receptor and OSM receptor leading to the activation of the JAK STAT and MAPK cascades.This review focuses on recent progress in the understanding of the molecular mechanisms of IL 6  [ **IL6** ]  type cytokine signal transduction.  Ref: 12773095 Biochem J< MedlineTA>Biochem J, 2003
- In this respect murine fibroblasts lacking exon 3 of SHP2 are not sensitive to TNF alpha, indicating that functional SHP2 and its recruitment to gp130  [ **IL6ST** ]  are key events in inhibition of IL 6  [ **IL6** ]  dependent STAT activation by TNF alpha.  Ref: 12817006 J Immunol, 2003
- We previously demonstrated that interleukin 6  [ **IL6** ]  related cytokine, leukemia inhibitory factor LIF, induced cardiac hypertrophy through gp130  [ **IL6ST** ] .  Ref: 12855672 Circ Res< MedlineTA>Circ Res, 2003
- The mRNAs of both receptors of IL 6  [ **IL6** ] , the soluble IL6R and gp130  [ **IL6ST** ] , were increased subsequently to IL 6  [ **IL6** ]  mRNA.  Ref: 12733076 Pflugers Arch< MedlineTA>Pflugers Arch, 2003

**Non-interactive relationship** (e.g. studied together, co-existance, homology, etc.)

- Here we show that phosphorylation of STAT3 is prolonged in mouse Socs3 deficient macrophages after stimulation with interleukin 6  [ **IL6** ]  IL 6  [ **IL6** ]  but not IL 10, indicating that SOCS3 specifically affects signaling mediated by IL 6  [ **IL6** ]  and gp130  [ **IL6ST** ] .  Ref: 12754506 Nat Immunol< MedlineTA>Nat Immunol, 2003
- To define the IL 6  [ **IL6** ]  dependent intracellular pathways more specifically, we generated a hepatocyte specific gp130  [ **IL6ST** ]  knockout mouse.  Ref: 12891556 Gastroenterology, 2003
- Interleukin 6  [ **IL6** ]  IL 6  [ **IL6** ]  activates cells by binding to the membrane bound IL 6  [ **IL6** ]  receptor IL 6R and subsequent formation of a gp130  [ **IL6ST** ]  homodimer.  Ref: 12832423 J Biol Chem< MedlineTA>J Biol Chem, 2003
- TNF alpha Induces Tyrosine Phosphorylation and Recruitment of the Src Homology Protein Tyrosine Phosphatase 2 to the gp130  [ **IL6ST** ]  Signal Transducing Subunit of the IL 6  [ **IL6** ]  Receptor Complex.  Ref: 12817006 J Immunol, 2003
- Furthermore, activation of p38 mitogen activated protein kinase is shown to be essential for the inhibitory effect of TNF alpha on IL 6  [ **IL6** ]  signaling and TNF alpha dependent recruitment of SHP2 to gp130  [ **IL6ST** ] .  Ref: 12817006 J Immunol, 2003
- Epidermal growth factor receptor independent constitutive activation of STAT3 in head and neck squamous cell carcinoma is mediated by the autocrine paracrine stimulation of the interleukin 6  [ **IL6** ]  gp130  [ **IL6ST** ]  cytokine system.  Ref: 12782602 Cancer Res, 2003
- ELISA was used to determine the serum levels of soluble interleukin 2 receptors, interleukin 1, interleukin 6  [ **IL6** ] , and 2 soluble interleukin 6  [ **IL6** ]  receptors sIL 6R and soluble gp130  [ **IL6ST** ]  in 18 patients with cluster headache 6 women and 12 men during the cluster period and in 17 healthy controls who were headache free 3 women and 14 men.  Ref: 12864761 Headache, 2003
- Interleukin 6  [ **IL6** ]  gp130  [ **IL6ST** ]  dependent pathways are protective during chronic liver diseases.  Ref: 12830005 Hepatology< MedlineTA>Hepatology, 2003
- Since recruitment of the tyrosine phosphatase Src homology protein tyrosine phosphatase 2 SHP2 to the signal transducing receptor subunit gp130  [ **IL6ST** ]  attenuates IL 6  [ **IL6** ]  mediated STAT activation, we were interested in whether TNF alpha also induces the association of SHP2 to the gp130  [ **IL6ST** ]  receptor subunit.  Ref: 12817006 J Immunol, 2003
- Fibroblasts Isolated from Normal Lungs and Those with Idiopathic Pulmonary Fibrosis Differ in Interleukin 6  [ **IL6** ]  gp130  [ **IL6ST** ]  Mediated Cell Signaling and Proliferation.  Ref: 12819039 Am J Pathol, 2003
- In this context the cytoplasmic SHP2 SOCS3 recruitment site of gp130  [ **IL6ST** ]  tyrosine 759 is shown to be important for the inhibitory effects of TNF alpha, since mutation of this residue completely restores IL 6  [ **IL6** ]  stimulated activation of STAT3 and, consequently, of a STAT3 dependent promoter.  Ref: 12817006 J Immunol, 2003
- To prove the relevance of these clinical findings, we applied the tetrachlorcarbonide CCl 4 model to conditional knockout animals Cre loxP system for gp130  [ **IL6ST** ] , the common signal transducer of IL 6  [ **IL6** ]  family cytokines.  Ref: 12830005 Hepatology< MedlineTA>Hepatology, 2003
- In macrophages lacking the Socs3 gene or carrying a mutation of the SOCS3 binding site in gp130  [ **IL6ST** ] , the lipopolysaccharide induced production of tumor necrosis factor TNF and IL 12 is suppressed by both IL 10 and IL 6  [ **IL6** ] .  Ref: 12754507 Nat Immunol< MedlineTA>Nat Immunol, 2003
- Cells that express gp130  [ **IL6ST** ] , but not the IL 6R, can be activated by IL 6  [ **IL6** ]  and the soluble IL 6R sIL 6R which is generated by shedding from the cell surface or by alternative splicing.  Ref: 12832423 J Biol Chem< MedlineTA>J Biol Chem, 2003
- BACKGROUNDLeukemia inhibitor factor LIF, a member of the interleukin 6  [ **IL6** ]  cytokine family, has recently been shown to inhibit HIV 1 replication both in vivo and in vitro.OBJECTIVELIF and its corresponding receptors gp130  [ **IL6ST** ]  and LIF receptor alpha LIFR alpha were studied in lymphoid tissue LT to reveal potential systemic immunoregulatory effects during the course of HIV 1 infection.METHODSLIF, gp130  [ **IL6ST** ] , LIFR alpha and HIV 1 replicating cells were identified at the single cell level by immunohistochemistry and quantified by computerized in situ imaging in tonsil and lymph nodes biopsies LT from patients with primary HIV 1 infection PHI, chronic HIV 1 infection cHI, long term non progressors LTNP and HIV 1 seronegative controls.RESULTSLIF and its receptors, gp130  [ **IL6ST** ]  and LIFR alpha were significantly P &lt.  Ref: 12799551 AIDS< MedlineTA>AIDS, 2003
- Hexameric structure and assembly of the interleukin 6  [ **IL6** ]  IL 6  [ **IL6** ]  alpha receptor gp130  [ **IL6ST** ]  complex.  Ref: 12829785 Science, 2003
- after IL 6  [ **IL6** ]  and OSM stimulation, gp130  [ **IL6ST** ]  dependent pathways STAT3, APR gene expression were completely blocked in the liver of these animals.  Ref: 12891556 Gastroenterology, 2003
